# Examination of the best head tilt angle to reduce the parotid gland dose maintaining a safe level of lens dose in whole‐brain radiotherapy using the four‐field box technique

**DOI:** 10.1002/acm2.13151

**Published:** 2021-01-10

**Authors:** Hidetoshi Shimizu, Koji Sasaki, Takahiro Aoyama, Hiroyuki Tachibana, Hiroshi Tanaka, Yutaro Koide, Tohru Iwata, Tomoki Kitagawa, Takeshi Kodaira

**Affiliations:** ^1^ Department of Radiation Oncology Aichi Cancer Center Hospital Nagoya Aichi Japan; ^2^ Graduate School of Radiological Technology Gunma Prefectural College of Health Sciences Maebashi Gunma Japan; ^3^ Graduate School of Medicine Aichi Medical University Nagakute Aichi Japan

**Keywords:** parotid gland, prophylactic cranial irradiation, small cell lung cancer, whole brain

## Abstract

The parotid gland is recognized as a major‐risk organ in whole‐brain irradiation; however, the beam delivery from the left and right sides cannot reduce the parotid gland dose. The four‐field box technique using a head‐tilting device has been reported to reduce the parotid gland dose by excluding it from the radiation field. This study aimed to determine the appropriate head tilt angle to reduce the parotid gland dose in the four‐field box technique. The bilateral, anterior, and posterior beams were set for each of ten patients. The orbitomeatal plane angle (OMPA) was introduced as an indicator that expresses the head tilt angle. Next, principal component analysis (PCA) was performed to understand the interrelationship between variables (dosimetric parameters of the lens and parotid gland and OMPA). In PCA, the angle between the OMPA vector and maximum lens dose or mean parotid gland dose vector was approximately opposite or close, indicating a negative or positive correlation [r = −0.627 (*p* < 0.05) or 0.475 (*p* < 0.05), respectively]. The OMPA that reduced the maximum lens dose to <10 Gy with a 95% confidence interval was approximately 14°. If the lens dose was not considered, the parotid gland dose could be reduced by decreasing the OMPA.

## 1 INTRODUCTION

After chemotherapy with or without thoracic radiotherapy for the limited disease small cell lung cancer (LD‐SCLC), brain recurrence occurred in 50–60% of patients with complete response in 3 yr.^1^ Currently, prophylactic cranial irradiation (PCI) is commonly used to prevent brain recurrence.^2^ Aupérin et al. revealed that PCI not only reduced brain recurrence rates but also provided a significant survival benefit in their meta‐analysis of 987 patients with complete response to SCLC.^3^ Yin et al. also indicated a positive role of PCI in improving survival and reducing the risk of brain metastases in patients with SCLC in their meta‐analysis of seven trials including 2114 patients.^4^ Thus, recognizing survival benefits of PCI for SCLC, side effects due to PCI must be reduced to improve the patients' quality of life.

In PCI, the beam is usually delivered from the left and right sides of the whole brain (WB). During an irradiation, the parotid gland is a major‐risk organ^5^, ^6^, ^7^, ^8^, ^9^, ^10^, ^11^, ^12^; however, the beam delivery from the left and right sides could not prevent the dose from entering the parotid glands. In fact, for patients who underwent palliative WB irradiation with the standard prescription of 30 Gy in 10 fractions, Noh et al. reported that 12 (37.5%) and 1 (3.1%) patients received the mean doses of >20 Gy or >25 Gy, respectively.^5^ This result indicated that some patients exceeded dose tolerances of the parotid glands, based on the report by Deasy et al. that tolerances of one parotid gland and both glands that effectively prevent severe xerostomia were the mean doses of 20 and 25 Gy, respectively.^13^ The standard prescription of PCI for LD‐SCLC is 25 Gy in 10 fractions,^14^ which is smaller than that for palliative cases (e.g., 30 Gy in 10 fractions). The risk of severe xerostomia may be relatively lower in patients who underwent PCI than in those who underwent palliative treatment; however, a lower mean dose to the parotid gland results in better function.^13^

Several researchers have suggested techniques to reduce the dose entering the parotid gland.^8^, ^11^, ^12^ Intensity‐modulated radiotherapy is one of the useful techniques; however, it is time‐consuming and costly. Regarding conventional techniques, Cho et al. reduced the parotid gland dose by cutting the radiation field at the patient’s foot side^8^; however, this technique is not suitable for patients with combined upper cervical spinal metastasis or leptomeningeal seeding. Using another technique, Park et al. reduced the parotid gland dose with a noncoplanar beam^11^; however, the technique is time‐consuming because of the couch rotation in the treatment room. Instead of using these techniques, they proposed a new technique that spared the parotid gland from radiation fields in the four‐field box irradiation using a head‐tilting device.^12^ This technique can be performed on patients with combined upper cervical spinal metastasis or leptomeningeal seeding and can be employed in many facilities because it is a simple four‐field box irradiation without noncoplanar beams. Therefore, this study aimed to determine the best head tilt angle to reduce the parotid gland dose in the four‐field box technique. The optimal head tilt angle should be determined to reduce the parotid gland dose while maintaining a safe level of lens dose. The risk of cataract progression is known to be dependent on the radiation dose. In the study by Merriam et al., it was reported that the risk of cataract progression after an irradiation of 2.5–6.5 Gy and 6.51–11.5 Gy is 33% (latency: 8 yr) and 66% (latency: 4 yr), respectively.^15^ Emami et al. estimated that the doses associated with the incidence of 5% and 10% of cataracts in 5 yr were 10‐ and 18‐Gy, respectively.^16^ Regarding the clinical goal of the lens dose in the treatment plan, Piroth et al.^17^ and Yamazaki et al.^18^ set 5 and 10 Gy at the maximum dose, respectively. These clinical goals were introduced as the dose constraint of the lens in accordance with a radiation oncologist’s guide.^19^ The four‐field box technique would be more practical after determining the optimal head tilt angle to reduce the parotid gland dose and maintain a safe level of lens dose.

## 2 MATERIALS AND METHODS

### 2.A Patient selection and imaging

Ten patients who had previously undergone WB irradiation were selected and immobilized from the top of the head to the chin with a thermoplastic mask without using a head‐tilting device, and 3‐mm slice‐thick computed tomography (CT) images (Aquilion LB, Cannon Medical Systems Co.) were acquired. In this study, instead of using a head‐tilting device, an approach that simulated the head tilt by a treatment planning system was adapted. This study was approved by our institutional review board (No. 2020‐1‐021).

### 2.B Contouring and treatment planning

The CT images were imported into a treatment planning system (RayStation, version 6.2.0, RaySearch Laboratories) for delineation of the target and high‐risk organ volumes. The WB was delineated as a clinical target volume (CTV). A planning target volume (PTV) was created by adding 5‐mm isotropic margins for the CTV. The parotid gland and lens were delineated as high‐risk organs.

**Fig. 1:**
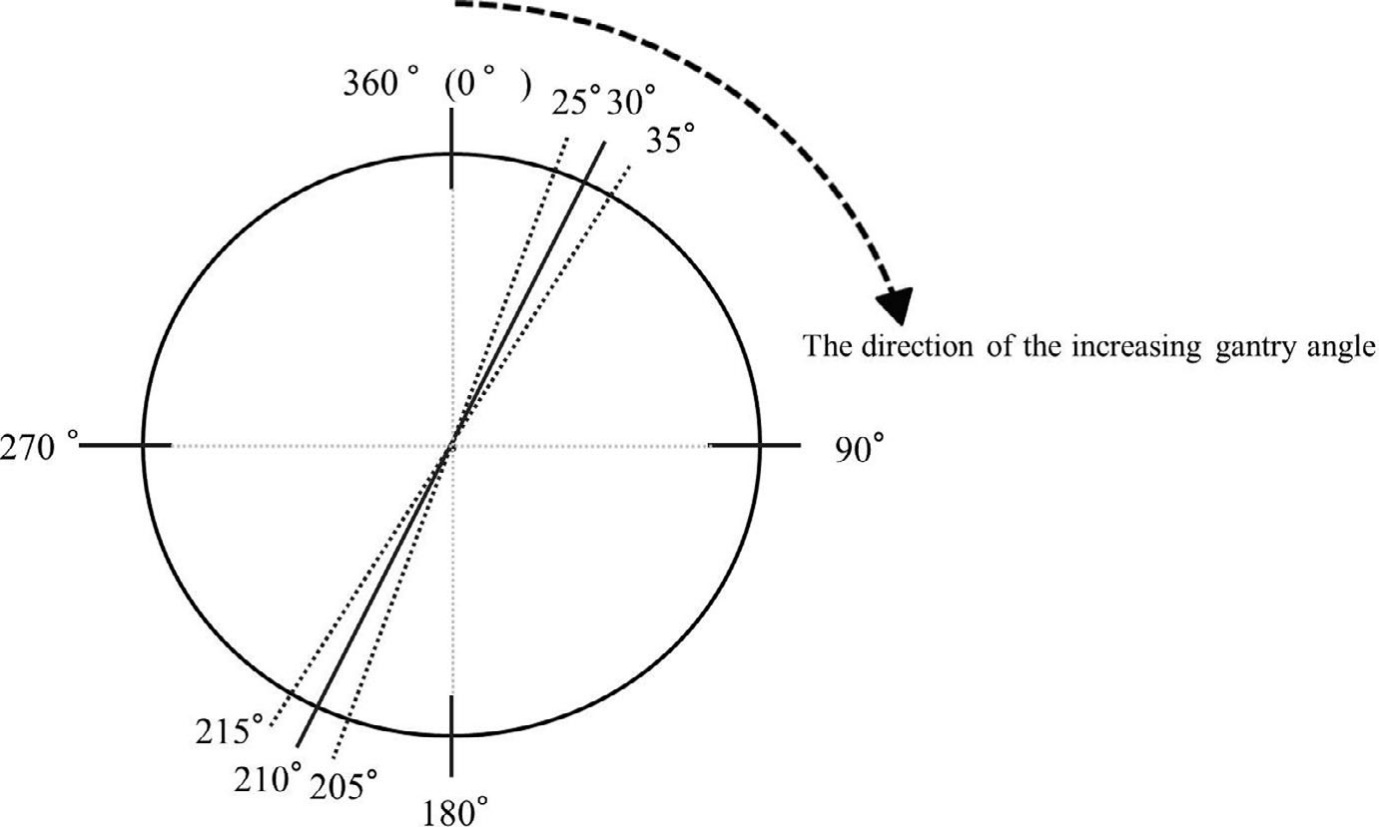
Definition of gantry angle. Opposite beam angles of 25°, 30°, and 35° were 205°, 210°, and 215°, respectively. The dashed arrow shows the direction of the increasing gantry angle.

First, conventional bilateral beams were set up so that the beamline of the anterior side was parallel to the line connecting the left and right eye sockets. Additionally, a beam from the anterior direction was added with the couch angle of 90°. The beam from the anterior direction was set at a gantry angle where the lens was not included in the irradiation field and the beam from the posterior direction was set on the opposite side. The four‐field box plan based on this procedure was defined as the “original plan.” Next, two new plans were created by tilting ±5° of the beam angle from the anterior and posterior directions of the original plan. For example, if beam angles from the anterior and posterior directions in the original plan were 30° and 210°, respectively: one was 35° and 205° and the other was 25° and 215° (Fig. 1). The remaining beams were unchanged.

**Fig. 2:**
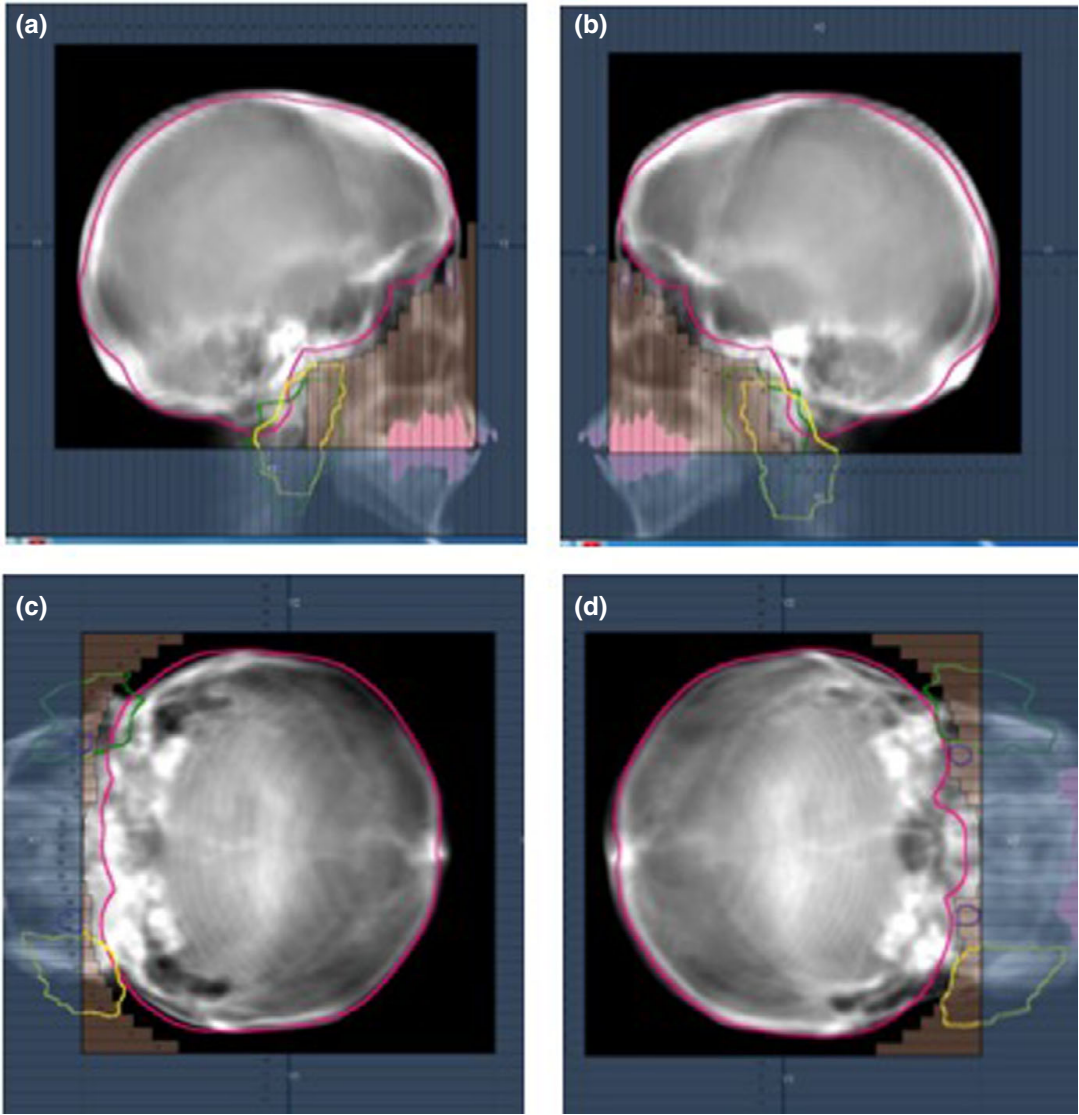
Representative radiation fields of the four‐field box technique; beams eye view from the (a) right, (b) left, (c) anterior, and (d) posterior directions. Pink, yellow, and purple contours show the planning target volume, parotid gland, and lens, respectively.

Regarding three plans for each patient, collimator angles of 90° and 0° were adapted for the bilateral and remaining beams, respectively. Although a leaf margin of ≥5 mm was basically added to the PTV, the multi‐leaf collimator was closed up to the position where it did not interfere the PTV in order to reduce the lens dose. Figure 2 shows representative radiation fields of the four‐field box technique.

The beam weight from each direction was equal, and no physical or virtual wedges were used. The energy of all beams was 10 MV of TrueBeam (Varian Medical Systems, Inc.). The PCI was assumed; therefore, a dose prescription of 25 Gy in ten fractions was used. The prescription to the reference point generally adopted in the WB irradiation greatly varies in dose distribution depending on the position. In this study, a volume prescription (prescription to 95% of the PTV: D_95%_) that facilitates easy planning comparison was adopted.

### 2.C Calculation of the head tilt angle and dosimetric parameters

**Fig. 3:**
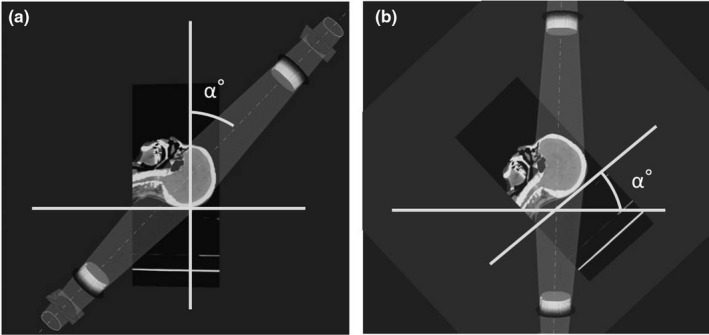
Head tilt simulations in the treatment planning system. Although a head‐tilting device was not used in the planned CT, the head tilt angle was set by measuring the gantry angle with the couch angle of 90°. For example, (a) the gantry angle of α° without the tilting device is equivalent to (b) the gantry angle of 0° with the tilting device of α°.

The head‐tilting device was not used in the treatment planning CT, and the head tilt angle was set by measuring the gantry angle with the couch angle of 90°. For example, (a) the gantry angle of α° without the tilting device is equivalent to (b) the gantry angle of 0° with the tilting device of α° as shown in Fig. 3. The virtual orbitomeatal plane angle (OMPA_virtual_) was introduced as an indicator that expresses the head tilt angle. It was defined by subtracting the actual OMPA in the direction perpendicular to the couch from the anterior gantry angle (A_gantry_):

(1) $$OMPA_{\text{virtual}}=A_{\text{gantry}}‐OMPA$$

The OMPA_virtual_ value increases when the patient’s jaw is pulled. Since the OMPA can be measure from outside the patient body without using CT images, by tilting the head for the calculated OMPA_virtual_ in the treatment planning CT phase, the above‐described treatment plan can be designed without rotating the couch.

To determine the best OMPA_virtual_ to reduce the parotid gland dose in a clinically usable treatment plan, the following parameters were calculated: the dose to 98% of the PTV (D_98%_), maximum dose, homogeneity index (HI)^20^ and conformity index (CI)^20^ of the PTV; mean and median doses, the volume receiving at least 5 Gy (V_5Gy_), and V_10Gy_ for the parotid gland; and maximum and mean doses, V_5Gy_, and V_10Gy_ for the lens. HI and CI values were calculated as shown in Appendix A.

### 2.D The interrelationship between dosimetric parameters and OMPA_virtual_ in the four‐field box plan

To understand the interrelationship between variables (dosimetric parameters and OMPA_virtual_), principal component analysis (PCA) was performed.^21^ PCA is a multivariate technique widely used in reducing dataset dimensionality to increase the interpretability while preserving as much information as possible. For that, PCA identifies a new set of uncorrelated variables (principal components, PCs) that result from linear combinations of the original ones and that successively maximize the variance. For example, the direction that maximizes the data variance from the gravity point of all data shows the first PC (PC1). Next, the direction that maximizes the data variance from the gravity point with respect to the direction perpendicular to PC1 is defined as the second PC (PC2). The direction of the maximum variance is repeatedly searched based on the number of dimensions of the original data. In PCA, the degree to which the PC explains data variation is expressed as a proportion. For example, when PC1 and PC2 proportions are 50% and 20%, respectively, the cumulative proportion is 70%, indicating that 70% of data variation can be explained using PC1 and PC2. In this study, PCs that achieved the cumulative proportion of 70% were used for data retention.^21^ In addition, a biplot expressed each data point corresponding to a treatment plan, and the variable represented by a vector was drawn. In the biplot, positively or negatively correlated variables have the angle of approximately 0° or 180° between vectors, respectively. Conversely, uncorrelated variables have an angle of approximately 90° between vectors.

### 2.E Calculation of normal tissue complication probability for parotid gland toxicity

Normal tissue complication probability (NTCP) was used to quantify the risk of xerostomia of the parotid glands related to the OMPA_virtual_. NTCP was calculated by applying the Lyman–Kutcher–Burman (LKB) model^22^, ^23^ with model parameters derived for the linear–quadratic equivalent doses at 2 Gy per fraction (EQD2); α/β = 3 Gy was assumed. The equivalent uniform dose showing the same radiobiological effect as the nonuniform dose received by the organ was used to calculate the NTCP, as shown in Appendix B. In this study, three different parameter combinations were used as shown in Appendix C.^24^, ^25^, ^26^

### 2.F Statistical analysis

The correlation between dosimetric parameters and OMPA_virtual_ in the four‐field box plan was analyzed using the Spearman’s rank correlation. The differences of dosimetric parameters and NTCPs among the plans with the head tilt of ±5° and the original plan were analyzed using Friedman rank‐sum test. Wilcoxon signed‐rank test as post‐hoc test with the Bonferroni’s method for multiple comparisons was used to further analyze the difference. All analyses were performed using R^TM^ statistical software (version 2.15.2; R Project for Statistical Computing; Vienna, Austria).^27^ *P* < 0.05 were considered to indicate significance.

## 3 RESULTS

### 3.A The interrelationship between dosimetric parameters and OMPA_virtual_ in the four‐field box plan

**Fig. 4:**
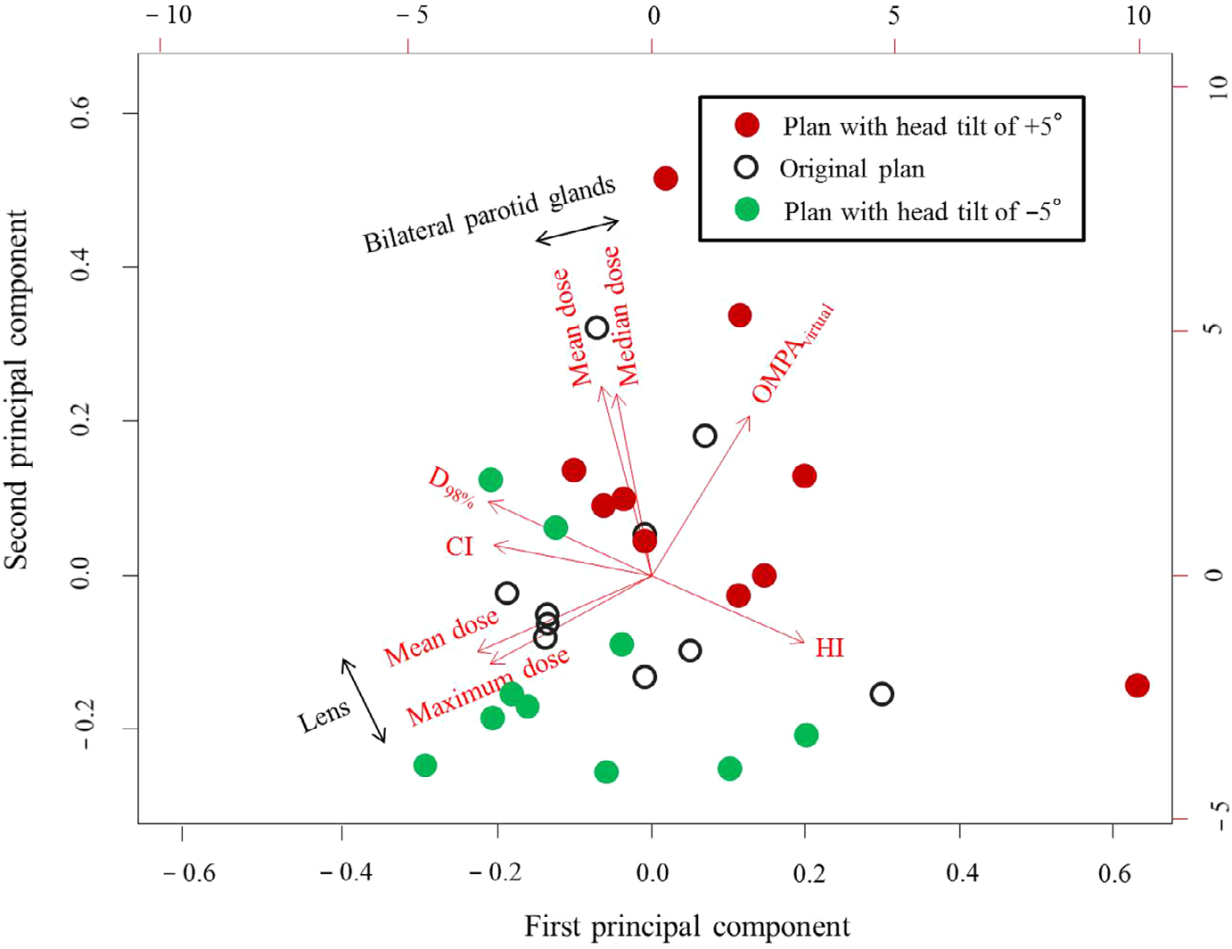
Biplot for the four‐field box plans. Data points indicated by black‐empty, red‐filled, and green‐filled circles correspond to the original plan, plan with head tilt of +5° to the original plan, and plan with head tilt of −5°, respectively. The variable is represented by a vector, where lateral and vertical axes of the secondary axis represent the weight of variable for the first and second principal components (PC1 and PC2), respectively. OMPA_virtual_, virtual orbitomeatal plane angle; D_98%_, the dose to volume of 98%; HI, homogeneity index; CI, conformity index.

In PCA, proportions of the PC1 and PC2 variance were 41% and 33%, respectively. The cumulative proportion was 74%. Figure 4 shows the biplot, where each data point corresponds to a treatment plan. The variable is also represented by a vector, where lateral and vertical axes of the secondary axis represent the weight of variables for the PC1 and PC2, respectively. The mean and maximum doses to the lens and the mean and median doses to the bilateral parotid glands had smaller angles between the vectors. Thus, two parameters of the lens and two of the bilateral parotid glands were strongly correlated with each other [Spearman’s rank correlation, r = 0.936 (*p* < 0.05) and 0.924 (*p* < 0.05), respectively]. Additionally, the angle between vectors of the OMPA_virtual_ and the mean or maximum dose to the lens was approximately 180°, which indicated a negative correlation [r = −0.591 (*p* < 0.05) or −0.627 (*p* < 0.05), respectively]. Furthermore, the angle between vectors of the OMPA_virtual_ and the mean or median dose to the bilateral parotid glands had a small value, which indicated a positive correlation [r = 0.475 (*p* < 0.05) or 0.405 (*p* < 0.05), respectively]. The OMPA_virtual_ was not correlated with the D_98%_, HI, or CI for the PTV [r = −0.118 (*p* = 0.535), 0.012 (*p* = 0.951), or 0.047 (*p* = 0.807), respectively], and the angle between vectors was approximately 90°. Regarding the distribution of data points, the data plan with the head tilt of +5° or −5° was located in the direction that increases the vector of the bilateral parotid gland doses or lens doses, respectively.

Dosimetric parameters and OMPA_virtual_ in each plan are provided in Table 1. The dose to the bilateral parotid glands (mean and median doses, V_5Gy_, and V_10Gy_) decreased with the decrease of the head tilt. In addition, the lens dose (maximum and mean doses, and V_5Gy_) increased with the decrease of the head tilt.

**Table 1:** The dosimetric parameters in the original plan and plans with head tilt of ±5°.

		(A) Original plan^a^	(B) Plan with head tilt of + 5°	(C) Plan with head tilt of − 5°	Freidman test	Wilcoxon signed rank test with Bonferroni's correction	
					*p*‐value	*p*‐value (A) vs (B)	*p*‐value (A) vs (C)
PTV	Maximum dose [Gy]	26.7 ± 17.5	26.7 ± 29.4	26.7 ± 17.7	0.47	–	–
	D_98%_ [Gy]	24.8 ± 0.2	24.7 ± 0.2	24.7 ± 0.2	0.23	–	–
	HI [%]	6.5 ± 1.4	7.3 ± 2.0	7.0 ± 1.2	0.49	–	–
	CI	0.8 ± 0.0	0.8 ± 0.0	0.8 ± 0.0	0.65	–	–
Lens	Maximum dose [Gy]	10.5 ± 2.0	8.5 ± 2.3	12.2 ± 2.0	<0.05	<0.05	<0.05
	Mean dose [Gy]	4.2 ± 0.6	3.6 ± 0.6	4.7 ± 0.6	<0.05	<0.05	<0.05
	V_5Gy_ [%]	19.8 ± 11.8	11.4 ± 10.7	30.9 ± 14.0	<0.05	<0.05	<0.05
	V_10Gy_ [%]	0.5 ± 0.9	0.2 ± 0.4	4.4 ± 9.1	<0.05	0.30	0.10
Bilateral parotid glands	Mean dose [Gy]	7.4 ± 1.8	8.2 ± 2.2	6.8 ± 1.5	<0.05	<0.05	<0.05
	Median dose [Gy]	5.6 ± 2.6	6.4 ± 3.0	5.2 ± 2.3	<0.05	<0.05	<0.05
	V_5Gy_ [%]	50.9 ± 9.1	53.7 ± 10.0	49.7 ± 8.9	<0.05	<0.05	<0.05
	V_10Gy_ [%]	34.5 ± 9.3	37.5 ± 9.7	32.9 ± 8.8	<0.05	<0.05	<0.05

PTV, planning target volume; D_98%_, the dose to volume of 98%; HI, homogeneity index; CI, conformity index; V_XXGy_, the volume receiving at least XX Gy.

^a^ Mean virtual orbitomeatal plane angle: 16 ± 2°.

**Fig. 5:**
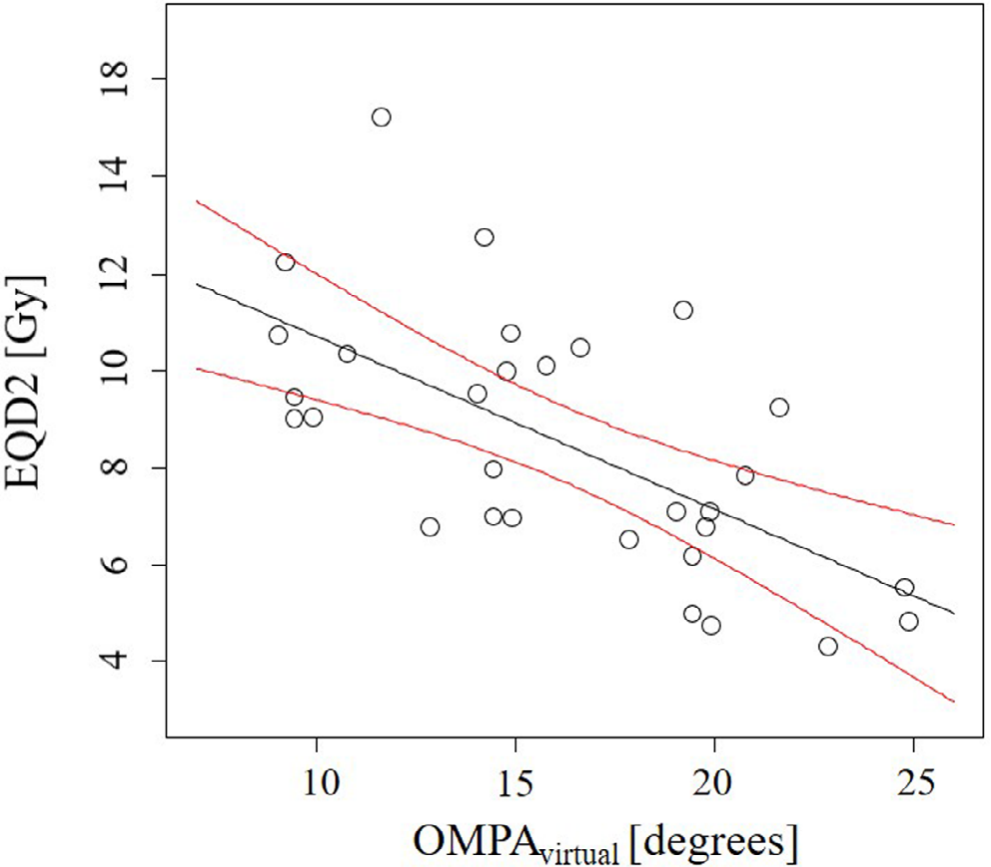
Relationships between the OMPA_virtual_ and maximum lens dose that converted to the linear–quadratic equivalent doses at 2 Gy per fraction (EQD2). The red line shows a 95% confidence interval. OMPA_virtual_, virtual orbitomeatal plane angle.

Figure 5 shows the relationships between OMPA_virtual_ and EQD2 converted from the maximum lens dose of our results. The red line shows a 95% confidence interval (CI). If the dose constraint of maximum lens dose was set at 10 Gy, the OMPA_virtual_ that reduced the maximum lens dose to <10 Gy with a 95% CI was approximately 14°.

### 3.B The dose distribution of a representative case

**Fig. 6:**
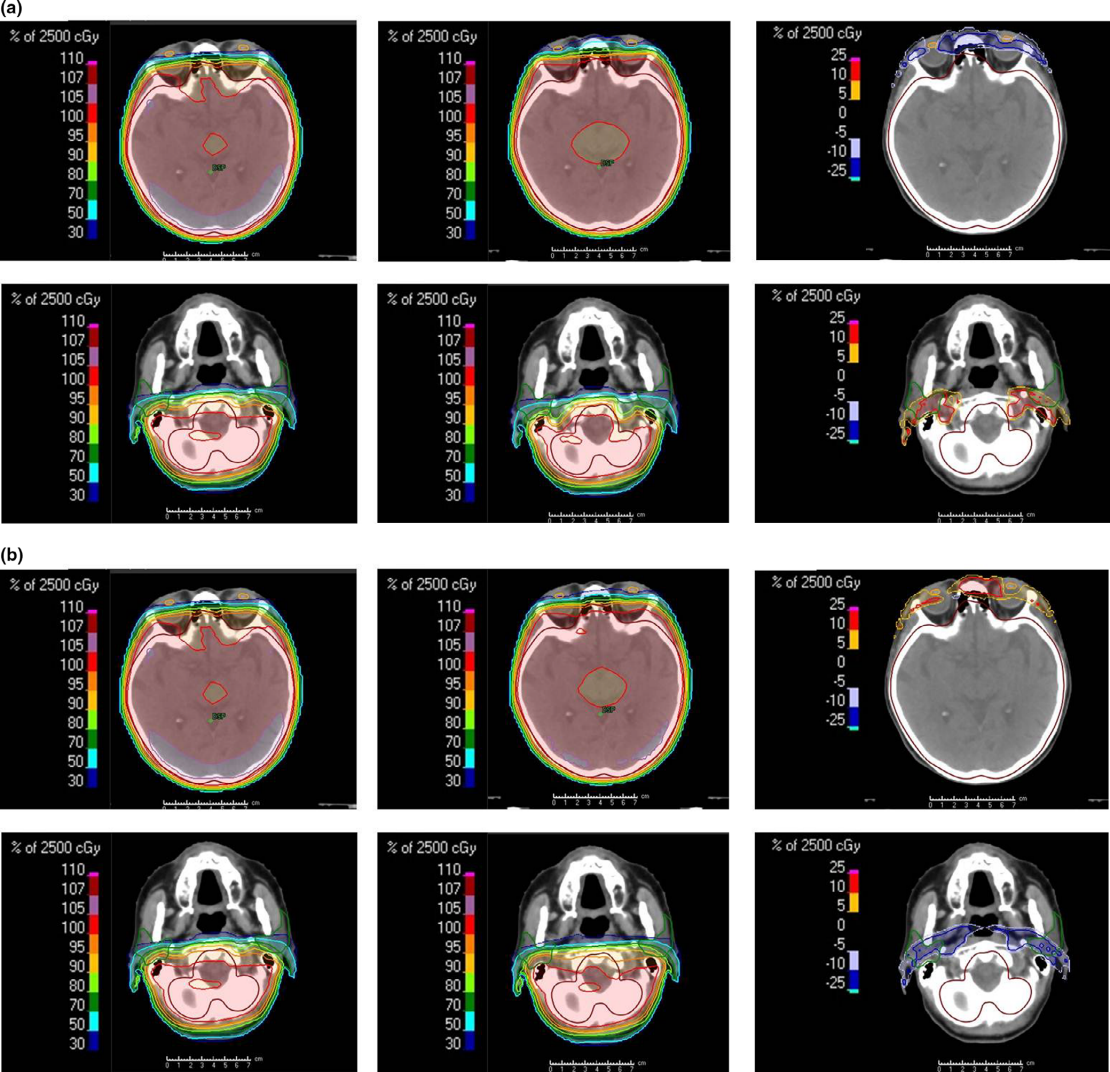
Above and below show the dose distribution of an axial plane in the lens and parotid gland level, respectively. (a) The left, middle and right figures show the dose distribution of the original plan, dose distribution of the plan with a head tilt of +5° to the original plan and imaging difference obtained by subtracting the original plan from the plan with a head tilt of +5°, respectively. (b) The left, middle and right figures show the dose distribution of the original plan, dose distribution of the plan with a head tilt of −5° and imaging difference obtained by subtracting the original plan from the plan with a head tilt of −5°, respectively.

Figure 6(a) shows the dose distribution in the axial plane at the lens and parotid gland levels in the CT image. The left, middle and right images show the dose distribution of the original plan, dose distribution of the plan with head tilt of +5° to the original plan and the imaging difference obtained by subtracting the original plan from the plan with head tilt of +5°, respectively. In the evaluation of the imaging difference, the dose around the lens decreased in the plan with head tilt of +5° than that in the original plan. In addition, the dose for the bilateral parotid glands in the plan with head tilt of +5° increased with compared to that in the original plan. The left, middle and right images in Fig. 6(b) show the dose distribution of the original plan, the dose distribution of the plan with head tilt of −5°, and the imaging difference obtained by subtracting the original plan from the plan with head tilt of −5°, respectively. According to the imaging difference, doses around the lens or bilateral parotid gland increased or decreased in the plan with head tilt of −5° than that of the original plan, respectively.

### 3.C Calculation of normal tissue complication probability for parotid gland toxicity

Table 2 shows the NTCP using three different parameter combinations. The NTCP was small in all plans. Excluding NTCP with parameters introduced by Eisbruch et al., the NTCP in the plan with the head tilt of +5° was significantly larger than that in the original plan (Wilcoxon signed‐rank test as *post‐hoc* test for Friedman rank‐sum test, *p* < 0.05). Additionally, NTCP in the plan with the head tilt of −5° was significantly smaller than that in the original plan (*p* < 0.05).

**Table 2:** Normal tissue complication probability using three different parameter combinations.

	(A) Original plan	(B) Plan with head tilt of +5°	(C) Plan with head tilt of −5°	Wilcoxon signed rank test with Bonferroni's correction *P *< 0.05
Burman et al. [24] [× 10^−5^]	0.35 ± 0.55	0.85 ± 1.40	0.18 ± 0.21	(A) vs (B)
				(A) vs (C)
				(B) vs (C)
Eisbruch et al. [25] [× 10^−4^]	0.31 ± 0.63	0.91 ± 2.13	0.11 ± 0.19	(A) vs (C)
				(B) vs (C)
Roesink et al. [26] [× 10^−1^]	0.32 ± 0.08	0.38 ± 0.12	0.30 ± 0.06	(A) vs (B)
				(A) vs (C)
				(B) vs (C)

## 4 DISCUSSION

In this study, the interrelationship between the OMPA_virtual_, an indicator of the head tilt angle and dosimetric parameter, was investigated to determine the best head tilt angle to reduce the parotid gland dose in WB radiotherapy using a four‐field box technique. In our results, the parotid gland dose decreased as the OMPA_virtual_ decreased, namely, with the elevation of the patient’s chin. Conversely, the lens dose increased with decreased OMPA_virtual_. These results indicated that decreased OMPA_virtual_ could not reduce both parotid gland and lens doses.

According to Fig. 5, if the dose constraint of maximum lens dose was set at 10 Gy, the OMPA_virtual_ that reduced the maximum lens dose to <10 Gy with a 95% CI was approximately 14°. This result indicated that the OMPA_virtual_ of 14° could decrease the parotid gland dose efficiently while maintaining the lens dose within a safe level. Conversely, if the lens of the eye can be replaced and the patient’s risk of cataracts does not need to be considered, the dose to the parotid gland could be further reduced by decreasing the OMPA_virtual_ (<14°). When the mean and median doses to the bilateral parotid glands were separated at the OMPA_virtual_ of 14°, both the mean and median doses to the bilateral parotid glands in the OMPA_virtual_ of <14° were smaller than those in the OMPA_virtual_ of >14° (data not shown, Wilcoxon rank‐sum test, *p* < 0.05).

In this study, the NTCP for the evaluation of parotid gland toxicity was calculated. Although three different parameter combinations (*n*, *m* and *D*_50_) were used, the NTCP with parameters reported from Roesink et al. was larger than that with others. Because *m* value reported from Roesink et al. is high, namely, the slope of the response curve is loose, the contribution of the low dose to the NTCP is also high. Therefore, the NTCP with parameters reported from Roesink et al. would be larger than that with others, in the low‐dose region treated in this study. However, the NTCP value was a small difference between the plan with the head tilt of +5° and that with the head tilt of −5°. In other words, the head tilt angle range used in this study had no influence on the occurrence of xerostomia in WB radiotherapy using a four‐field box technique. On the other hand, Deasy et al. reported that the mean dose of <10 Gy to the parotid gland results in better function.^13^ In our results, the proportion of bilateral parotid glands that received the mean dose of ≥10 Gy were 15% in the OMPA_virtual_ of >14° and 0% in the OMPA_virtual_ <14°. Thus, although the difference between NTCPs was not clinically significant, it is expected to reduce xerostomia by adapting the best head tilt angle.

Regarding the dosimetric parameter in WB radiotherapy using a four‐field box technique, Park et al. reported that the maximum dose to the lens and the mean dose to the bilateral parotid glands were approximately 5 and 5.8 Gy, respectively.^12^ In our results, as shown in Table 1, they were 10.5 and 7.4 Gy, respectively. Compared to the previous studies, the values in this study were higher. The D_95%_ prescription was used to facilitate comparison in this study, resulting in higher doses than the point prescription. In addition, especially for the lens, it would be caused by the dose coverage of the PTV having been prioritized when the PTV overlapped with the lens. If the standard point prescription was adapted in the treatment planning, the best head tilt angle to protect the lens and reduce the parotid gland dose would be different from the OMPA_virtual_ of 14°. A point prescription generally makes the overall dose including the lens dose smaller than that of the D_95%_ prescription; therefore, the lens could be protected by the OMPA_virtual_ of <14°. In a point prescription, the OMPA_virtual_ of 14° would lead to low risk of cataract development.

The prescription dose of the PCI assumed in this study is smaller than that of the palliative WB irradiation (typically 30 Gy in ten fractions). In palliative WB irradiation, the best head tilt angle would be larger than that of this study (the OMPA_virtual_ of 14°). Therefore, adaptation of the OMPA_virtual_ of 14° would likely exceed the maximum lens dose of 10 Gy. If the lens needs to be protected, the OMPA_virtual_ of 14° should not be adapted.

This study had some limitations. First, the head tilt is simulated during the treatment planning without a head‐tilting device. Therefore, some discrepancies might occur in the actual treatment planning using a head‐tilting device. Next, the NTCP value was slightly different between the plans with a head tilt. In order to clarify the efficacy of parotid gland preservation using the head tilt, whether the parotid gland function is preserved should be investigate in clinical trials. Finally, datasets from ten patients used in this study might be slightly small to improve the maturity of the evaluation of this study.

## 5 CONCLUSION

This study examined the best head tilt angle to reduce the parotid gland dose in WB radiotherapy using a four‐field box technique. Since the parotid gland dose is inversely related to the lens dose, the orbitomeatal plane angle required to reduce the maximum lens dose to ≤10 Gy and minimize the parotid gland dose was 14°. If the lens dose was not considered, the parotid gland dose could be reduced by decreasing the orbitomeatal plane angle.

## AUTHOR CONTRIBUTION

Hidetoshi Shimizu conceived the study, performed analysis and interpretation of data, and drafted the manuscript, supported by Koji Sasaki. Koji Sasaki, Takahiro Aoyama, Hiroyuki Tachibana, Hiroshi Tanaka, Yutaro Koide, Tohru Iwata, Tomoki Kitagawa, and Takeshi Kodaira were involved in the study design and contributed significantly to the editing of the manuscript. All authors read and approved the final manuscript.

## CONFLICT OF INTEREST

No conflict of interest to declare.

## ETHICAL APPROVAL

This study was approved by the Institutional Review Board of our hospital (No. 2020‐1‐021).

## Appendix A

HI and CI values were calculated as follows:

(A1) $$\text{HI}=\frac{D_{2\%}‐D_{98\%}}{D_{50\%}}\times 100\left[\%\right],$$

(A2) $$\text{CI}=\frac{TV_{\text{RI}}}{\mathrm{TV}}\times \frac{TV_{\text{RI}}}{V_{\text{RI}}}.$$

In Eq. (A1), D_2__%_, D_98%_ and D_50%_ indicated the doses of 2%, 98% and 50% of the target volume, respectively. In Eq. (A2), *TV* was the target volume, *TV*_RI_ was the target volume covered by the reference isodose line, and *V*_RI_ was the volume inside the reference isodose line. In this study, the reference isodose was 25 Gy.

## Appendix B

Normal tissue complication probability (NTCP) was calculated using the following equation:

(B1) $$NTCP=\frac{1}{\sqrt{2\pi }}\int_{‐\infty }^{t}e^{\frac{‐x^{2}}{2}}dx.$$

where

(B2) $$t=\frac{EUD‐D_{50}}{m\cdot D_{50}},$$

and

(B3) $$EUD=\sum_{i=1}^{M}{\left(\frac{v_{i}}{v_{\text{ref}}}EQD2_{i}^{1/n}\right)}^{2}.$$

Parameters (*n*, *m* and *D*_50_) show a factor that controls the volume effect, slope of the response curve, and dose providing a 50% response probability, respectively. *M*, *v_i_* and *v*_ref_ values show the total number of voxels, volume of voxel *i* and the reference volume, respectively.

## Appendix C

Parameters for Lyman–Kutcher–Burman model

**Table:**

LKB model parameters			
*n*	*D*_50_ [Gy]	*m*	End‐point
0.70	46.00	0.18	Total xerostomia^24^
1.00	28.40	0.18	25% xerostomia at 1 yr^25^
1.00	39.00	0.45	25% xerostomia at 1 yr^26^

LKB, Lyman–Kutcher–Burman; *n*, a factor that controls the volume effect; *m*, slope of the response curve; *D*_50_, the dose giving a 50% response probability.
